# Coping with the calcium overload caused by cell injury: ER to the rescue

**DOI:** 10.15698/cst2021.05.249

**Published:** 2021-04-16

**Authors:** Goutam Chandra, Davi A. G. Mázala, Jyoti K. Jaiswal

**Affiliations:** 1 Center of Genetic Medicine Research, Children's National Research Institute, 111 Michigan Av NW, Washington, DC 20010, Washington, DC.; 2 Inter University Centre for Biomedical Research & Super Specialty Hospital, Mahatma Gandhi University Campus at Thalappady, Kottayam, Kerala, India.; 3 Department of Kinesiology, College of Health Professions, Towson University, Maryland, U.S.A.; 4 Department of Genomics and Precision Medicine, George Washington University School of Medicine and Health Sciences, Washington, DC.

**Keywords:** endoplasmic reticulum, plasma membrane, anoctamin, TMEM16, limb girdle muscular dystrophy, ion channel, membrane repair

## Abstract

Cells maintain their cytosolic calcium (Ca^2+^) in nanomolar range and use controlled increase in Ca^2+^ for intracellular signaling. With the extracellular Ca^2+^ in the millimolar range, there is a steep Ca^2+^ gradient across the plasma membrane (PM). Thus, injury that damages PM, leads to a cytosolic Ca^2+^ overload, which helps activate PM repair (PMR) response. However, in order to survive, the cells must cope with the Ca^2+^ overload. In a recent study (Chandra *et al.* J Cell Biol, doi: 10.1083/jcb.202006035) we have examined how cells cope with injury-induced cytosolic Ca^2+^ overload. By monitoring Ca^2+^ dynamics in the cytosol and endoplasmic reticulum (ER), we found that PM injury-triggered increase in cytosolic Ca^2+^ is taken up by the ER. Pharmacological inhibition of ER Ca^2+^ uptake interferes with this process and compromises the repair ability of the injured cells. Muscle cells from patients and mouse model for the muscular dystrophy showed that lack of Anoctamin 5 (ANO5)/Transmembrane protein 16E (TMEM16E), an ER-resident putative Ca^2+^-activated chloride channel (CaCC), are poor at coping with cytosolic Ca^2+^ overload. Pharmacological inhibition of CaCC and lack of ANO5, both prevent Ca^2+^ uptake into ER. These studies identify a requirement of Cl^-^ uptake by the ER in sequestering injury-triggered cytosolic Ca^2+^ increase in the ER. Further, these studies show that ER helps injured cells cope with Ca^2+^ overload during PMR, lack of which contributes to muscular dystrophy due to mutations in the ANO5 protein.

It was nearly a century ago that Lewis Victor Heilbrunn identified the requirement of extracellular Ca^2+^ for what he termed as ‘*surface precipitation reaction*', a process that allows the cytoplasm streaming out from an injured cell to coagulate. Studies since then have identified that entry of extracellular Ca^2+^ into the injured cells trigger vesicle fusion, membrane remodeling, and cytoskeletal reorganization, all of which are required to repair the PM of the injured cell. While it is well recognized that excess cytosolic Ca^2+^ causes cell death, there is little understanding of how cells handle the large amount of extracellular Ca^2+^ that enters the injured cell. Being the largest membrane bound organelle and the major site for storing intracellular Ca^2+^, the ER is likely to aid in coping with the cytosolic Ca^2+^ overload. In a recent study, we used an ER-targeted Ca^2+^ sensor and found that ER responds to PM injury by rapidly sequestering the extracellular Ca^2+^ that enters the injured cell. This uptake of Ca^2+^ by the ER was mediated by the sarco/endoplasmic reticulum Ca^2+^-ATPase (SERCA) – the ER resident pump required for cytosolic Ca^2+^ uptake into the ER. Inhibition of SERCA activity in injured cells through a pharmacological inhibitor (thapsigargin), prevented ER Ca^2+^ uptake. Concomitantly, this also impaired the ability of the injured cells to sequester the cytosolic Ca^2+^, and impaired PMR. While this identified the role of ER in the injured cell's ability to cope with Ca^2+^ influx, it raised two additional questions:

1. What is the mechanism by which ER senses and buffers increased cytosolic Ca^2+^?
2. Why does failure to buffer cytosolic Ca^2+^ result in poor repair of the injured cell?

The answer to the first question is confounded by the fact that SERCA activity is not regulated by the cytosolic Ca^2+^ level. Instead, sustaining SERCA-mediated ER Ca^2+^ uptake requires the lumen of the ER to be kept electroneutral through the export of H^+^ and import of negatively charged ions such as Chloride (Cl^−^). Previously, we had observed that cells from Limb Girdle Muscular Dystrophy 2L (LGMD2L) patients, repair poorly due to lack of an ER-localized putative Ca^2+^ activated chloride channel (CaCC) protein - Anoctamin 5 (ANO5) also called Transmembrane 16E (TMEM16E). If injury-triggered increase in cytosolic Ca^2+^ activates ANO5-mediated pumping of Cl^-^ ions into the ER, then this could be the mechanism for the activation of Ca^2+^ uptake by the ER in healthy cells. In our recent study we found that similar to thapsigargin-treated healthy cells, LGMD2L patient cells are also poor at coping with injury-triggered increase in the cytosolic Ca^2+^. Through the use of a ER-targeted anion sensor, our study identified that PM injury led to anion uptake into the ER, which is compromised by NPPB (5-nitro-2-(3-phenylpropyl-amino) benzoic acid) - an inhibitor of CaCC. Similar to NPPB treated healthy cells, anion uptake into the ER was compromised in the injured LGMD2L patient cells. Concomitantly, the ER of the NPPB treated healthy cells and of the LGMD2L patient cells were both compromised in their ability to uptake injury-triggered increase in cytosolic Ca^2+^. By using a ANO5 knockout mouse, we examined Ca^2+^ homeostasis in injured ANO5-deficient muscle fibers, which identified that ANO5-deficient fibers are compromised in their ability to cope with the injury-triggered Ca^2+^ overload and in repairing PM damaged by focal injury or mechanical activity. Together, above results identify that cytosolic Ca^2+^ increase in injured cells triggers ER-resident CaCC activity, which supports clearance of cytosolic Ca^2+^ by the ER (**Figure 1**). This mechanism is the basis for the ER Ca^2+^ uptake in the injured cells and ANO5/TMEM16E is the CaCC that supports ER-mediated clearance of cytosolic Ca^2+^ overload in injured muscle cells.

**Figure 1:**
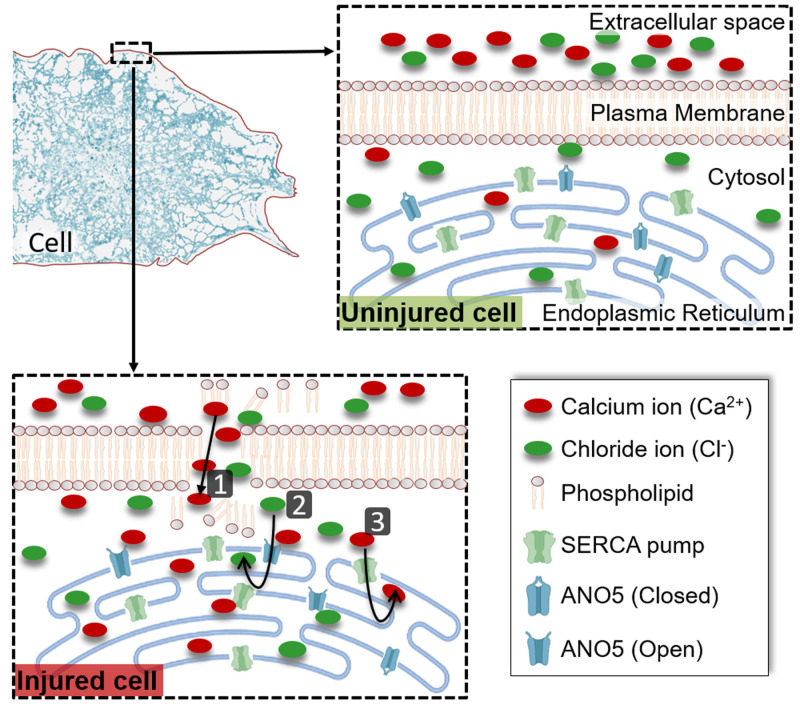
FIGURE 1: ER-mediated buffering of injury-triggered cytosolic Ca^2+^ increase. Plasma membrane acts as a barrier to maintain cytosolic ion balance, which is disrupted by cell injury leading to influx of ions such as Ca^2+^ (1). This increase in cytosolic Ca^2+^ activates opening of the ER-resident ion channel ANO5 and increases influx of the Cl^-^ ions into the ER lumen (2). This Cl^-^ ion serves as the counterion to maintain ER electroneutrality as the excess cytosolic Ca^2+^ is pumped into the ER lumen by the ER-resident Ca^2+^ pump SERCA (3). Together, these transporters enable buffering the cytosolic Ca^2+^ overload caused by PM injury, and their inhibition prevents clearance of cytosolic Ca^2+^ increase in the injured cell, compromising repair of the injured cell.

Given the requirement of Ca^2+^ increase for PMR, the next question was why does failure to rapidly buffer cytosolic Ca^2+^ compromise the ability of the injured cell to repair. The answer came from examining (A) Annexin-mediated PMR, and (B) Mitochondria-mediated PMR. Based on the Ca^2+^ affinity of the different annexins, PM injury causes sequential recruitment and clearance of annexins at the injury site. This injury-triggered dynamics of annexins is required for remodeling and repair of the injured PM. ANO5 deficit in the LGMD2L patient cells altered the injury-triggered dynamics of two of the annexin proteins we examined - Annexin A1 and Annexin A2. While both these annexins were recruited to the site of injury in patient cells at the kinetics similar to the healthy cells, prolonged cytosolic Ca^2+^ increase in patient cells correlated with the failure of annexins to clear from the injury site, interfering with annexin-mediated PMR. Another PMR pathway is mediated by the injury-proximal mitochondria, which helps clear some of the cytosolic Ca^2+^, but also get depolarized and increases reactive oxygen species (ROS) production. ROS from these mitochondria then activates local buildup of actin cytoskeleton that helps close the PM wound. Injury of patient cells lacking ANO5 increased Ca^2+^ uptake and caused extensive depolarization of their mitochondria, impairing mitochondria-mediated PMR. Based on these findings we concluded that failure of ER to sequester injury-triggered increase in cytosolic Ca^2+^ impairs spatial and temporal regulation of PMR response, which compromises the ability of these cells to repair. This indicated that approaches to restore cytosolic Ca^2+^ balance in the injured ANO5 deficient cells could improve their repair ability. Accordingly, our study identified improved repair of ANO5 knockout muscle fibers treated with a cell permeant Ca^2+^ chelator - BAPTA-AM (1,2-bis(o-aminophenoxy) ethane-N,N,N9,N9-tetraacetic acid-acetoxy-methyl ester).

Together, these results highlight the Goldilocks-like nature of injury-triggered Ca^2+^ increase, such that too little or too much increase both compromise PMR. It shows that ER-mediated cytosolic Ca^2+^ uptake allows the control over the amplitude and time of cytosolic Ca^2+^ increase in the injured cells. It highlights that Ca^2+^ uptake by the ER in injured cell relies upon Ca^2+^-activated anion transport and failure of anion or Ca^2+^ uptake by the ER disrupts the fine balance of multiple PMR repair responses mounted by the cell, compromising the cell's repair ability. Identification of this ER-mediated Ca^2+^ and PMR regulation not only identifies a new role for this organelle, but also offer insights into how defect in a putative anion channel can cause poor muscle repair in LGMD2L patients and point to future avenues for its therapy.

